# CAR-T-cell therapy for autoimmune neurologic diseases

**DOI:** 10.3389/fimmu.2026.1942249

**Published:** 2026-10-12

**Authors:** Yunhan Zhao, Yunyu Ding, Congfeng Tang, Zhengrong Wei, Yuqing Fan, Xianhui Hu, Jilong Yi

**Affiliations:** 1 Department of Neurology, Jingmen Central Hospital, Jingmen, Hubei, China; 2 Department of Neurology, Jingmen Central Hospital Affiliated to Jingchu University of Technology, Jingmen, Hubei, China; 3 Department of Nursing, Jingmen Central Hospital, Jingmen, Hubei, China; 4 Department of Cardiology, Jingmen Central Hospital Affiliated to Jingchu University of Technology, Jingmen, Hubei, China

**Keywords:** autoimmune neurologic disease, B cells, BCMA, CAR-T cells, MOGAD, multiple sclerosis, myasthenia gravis, neuroimmunology

## Abstract

Autoimmune neurologic diseases include multiple sclerosis, neuromyelitis optica spectrum disorder, myelin oligodendrocyte glycoprotein antibody-associated disease, autoimmune encephalitis, myasthenia gravis, stiff-person syndrome, chronic inflammatory demyelinating polyradiculoneuropathy, and related syndromes. The contributions of B cells, plasma cells, and autoantibodies differ substantially across these disorders; however, the B-cell lineage represents a therapeutically actionable target in many of them. Chimeric antigen receptor T-cell therapy, originally developed for hematologic malignancies, offers a potential strategy for deep and potentially durable depletion of pathogenic B-lineage cells and subsequent immune reconstitution. In recent years, CD19, BCMA, dual-target, mRNA CAR-T-cell, and chimeric autoantibody receptor T-cell approaches have generated preliminary clinical or translational evidence, primarily in systemic autoimmune diseases. Clinical evidence in neurologic disorders remains limited and spans myasthenia gravis, NMOSD, MOGAD, multiple sclerosis, autoimmune encephalitis, stiff-person syndrome, and CIDP. This review summarizes the biologic rationale, disease-specific evidence, target selection, safety concerns, and trial-design considerations for CAR-T-cell therapy in autoimmune neurologic diseases. Current evidence supports further investigation of CAR-T cells in selected patients with highly refractory, antibody- or B-cell-driven neuroimmune disorders, but does not establish superiority over existing therapies. However, the field remains dominated by case reports and small early-phase studies, with randomized evidence currently confined to one phase 2b trial in myasthenia gravis. Establishing the risk-benefit profile will require stringent patient selection, disease-specific neurologic monitoring, controlled prospective studies, and long-term follow-up.

## 1 Introduction

Chimeric antigen receptor T-cell (CAR-T-cell) therapy genetically redirects T cells to recognize, activate against, and eliminate antigen-expressing target cells. Its success in B-cell malignancies is based on two core features: direct surface-antigen recognition without conventional HLA restriction and the capacity of infused cells to expand, migrate, and persist after administration (1). Autoimmune neurologic diseases are not a single entity but a group of syndromes in which B cells, T cells, complement, innate immunity, antibodies, and tissue-local inflammation contribute in different proportions. Conventional immunotherapies and monoclonal antibodies have substantially improved outcomes, yet relapse, incomplete tissue immune-cell depletion, chronic immunosuppressive burden, and disease rebound after treatment withdrawal remain major clinical problems.

B cells contribute to autoimmunity through more than antibody production. They also act as antigen-presenting cells, secrete cytokines, shape ectopic lymphoid structures, and modulate T-cell responses. The differential effects of anti-CD20, anti-CD19, and plasma-cell-directed therapies indicate that distinct stages of B-cell development may be relevant in different diseases (2). In central nervous system disease, B cells may reside in cerebrospinal fluid, meninges, perivascular spaces, and parenchymal lesions; their anatomic and phenotypic diversity means that peripheral blood measures incompletely capture tissue inflammation (3). Thus, the central question for CAR-T-cell therapy in neuroimmunology is not simply whether B cells can be depleted. It is whether the relevant pathogenic cell populations can be depleted in the right patients, at the right time, and with acceptable risk.

The feasibility of CAR-T-cell therapy for autoimmunity was first supported by lupus models and clinical studies. CD19-directed CAR-T cells produced sustained B-cell depletion and ameliorated disease in murine lupus (4). Subsequently, patients with refractory systemic lupus erythematosus achieved clinical remission and autoantibody reduction without ongoing immunosuppression after CD19-directed CAR-T-cell therapy (5). A longer follow-up case series across autoimmune diseases further supported the hypothesis of deep B-cell depletion, regeneration of a renewed B-cell repertoire, and immune resetting (6). Tissue-level data also suggest that CD19-directed CAR-T cells can deplete B cells beyond peripheral blood, including in deep tissues (7). These findings cannot be directly extrapolated to neurologic diseases, but they provide a rational biologic foundation for neuroimmunologic exploration. A systematic review of CAR-T-cell therapy for autoimmune diseases of the central nervous system has similarly emphasized that the evidence base is expanding rapidly but remains early (8). The biologic logic linking pathogenic B-lineage immunity to target selection and subsequent immune reconstitution is summarized in **Figure 1**.

**Figure 1:**
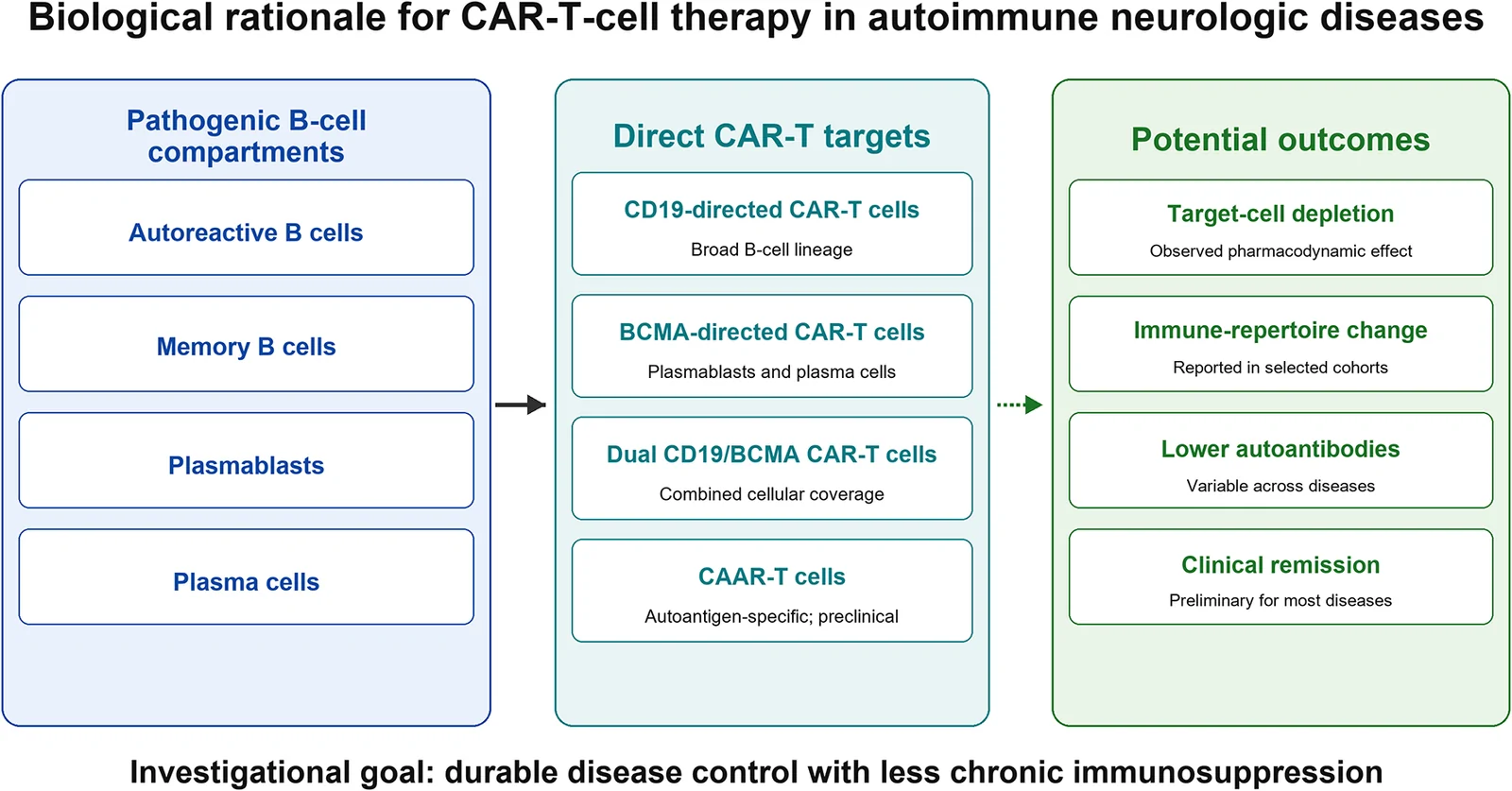
Biological rationale for CAR-T-cell therapy in autoimmune neurologic diseases. The schematic links pathogenic B-lineage compartments (autoreactive and memory B cells, plasmablasts, and plasma cells) to CD19-directed, BCMA-directed, dual CD19/BCMA-directed, and CAAR-T strategies. Reported or proposed downstream effects are presented according to evidence maturity: target-cell depletion is an observed pharmacodynamic effect, immune-repertoire change has been described in selected cohorts, reductions in autoantibody levels are variable, and clinical remission remains preliminary.

### 1.1 Literature search and evidence selection

This narrative review draws on targeted searches of PubMed-indexed literature, updated during revision in September 2026 without a fixed lower publication-date limit. Searches combined CAR-T-cell or chimeric antigen receptor terms with the names of the neurologic diseases covered in this review; CAAR-T, CD19, and BCMA terms were used for platform-specific searches. Clinical evidence was selected for direct relevance to autoimmune neurologic disease and the availability of reported patient outcomes, prioritizing peer-reviewed prospective trials and including case reports and small series where higher-level evidence was unavailable. Preclinical studies, systemic-autoimmunity studies, and selected reviews were used for mechanistic and safety context and were distinguished from direct neurologic clinical evidence. Study design, sample size, comparator availability, follow-up, and safety reporting guided interpretation. The synthesis is narrative and does not claim exhaustive study identification or quantitative pooling.

## 2 Mechanistic rationale and target selection

A conventional CAR comprises an extracellular antigen-recognition domain, a hinge or spacer, a transmembrane domain, and intracellular activation and costimulatory domains. Most second-generation CARs combine CD3ζ signaling with CD28 or 4-1BB costimulation (1, 9). Recent modifications include a second or tandem CAR, calibrated signaling domains that alter cytokine production and persistence, transient mRNA expression, and investigational *in situ* CAR-T-cell generation using targeted delivery systems (9–13). *In situ* generation remains a preclinical technology outside neuroautoimmunity.

CAR-T-cell strategies for autoimmune neurologic diseases can be grouped into four broad categories. The first is CD19-directed CAR-T-cell therapy, which targets a broad B-cell lineage from precursor B cells through plasmablasts and may deplete populations that are incompletely reached by CD20-directed treatment. The second is BCMA-directed CAR-T-cell therapy, which targets plasmablasts and plasma cells and is therefore particularly relevant when sustained autoantibody production is thought to be maintained by antibody-secreting cells. The third is dual CD19/BCMA-directed or sequential targeting, designed to address both antigen-presenting B cells and antibody-secreting cells. The fourth includes chimeric autoantibody receptor T cells (CAAR-T cells) and transient mRNA CAR-T-cell approaches. CAAR-T cells aim to selectively eliminate autoreactive B cells, whereas mRNA CAR-T cells emphasize controllability and repeat dosing while potentially reducing risks associated with long-term CAR-T persistence (10, 11, 14–18).

Compared with monoclonal antibodies targeting the same antigen or an adjacent B-lineage compartment, CAR-T cells can expand after antigen recognition, serially eliminate target cells, migrate into tissues, and persist after infusion. These properties may permit deeper and more durable depletion of pathogenic B-cell populations, including cells incompletely reached by circulating antibodies (6, 7). However, no controlled study has established the superiority of CAR-T-cell therapy over monoclonal-antibody therapy in an autoimmune neurologic disease. Any potential benefit must therefore be balanced against lymphodepletion, manufacturing complexity, cytokine release syndrome, neurotoxicity, hypogammaglobulinemia, and infection.

Target selection in neuroimmunology should be driven by pathophysiology rather than diagnostic labels alone. Diseases in which pathogenic antibodies, complement activation, or antibody titers closely track activity may be more suitable for B-cell or plasma-cell targeting. In contrast, disease phases dominated by compartmentalized central inflammation, myeloid activation, or neurodegeneration are unlikely to be reversed by B-cell depletion alone (2, 3). Lesion accessibility is another crucial issue. The central nervous system contains the blood-brain barrier, cerebrospinal fluid circulation, and meningeal immune niches; peripheral nerves and neuromuscular junctions involve blood-nerve barriers and antibody effects at synaptic or nodal structures. The migration, expansion, and persistence of CAR-T cells in these compartments require disease-specific study. A practical target-selection framework across the B-cell lineage is shown in **Figure 2**. To clarify how the current evidence differs across disease entities, **Table 1** summarizes candidate targets, evidence status, and key interpretive cautions for major autoimmune neurologic diseases.

**Figure 2:**
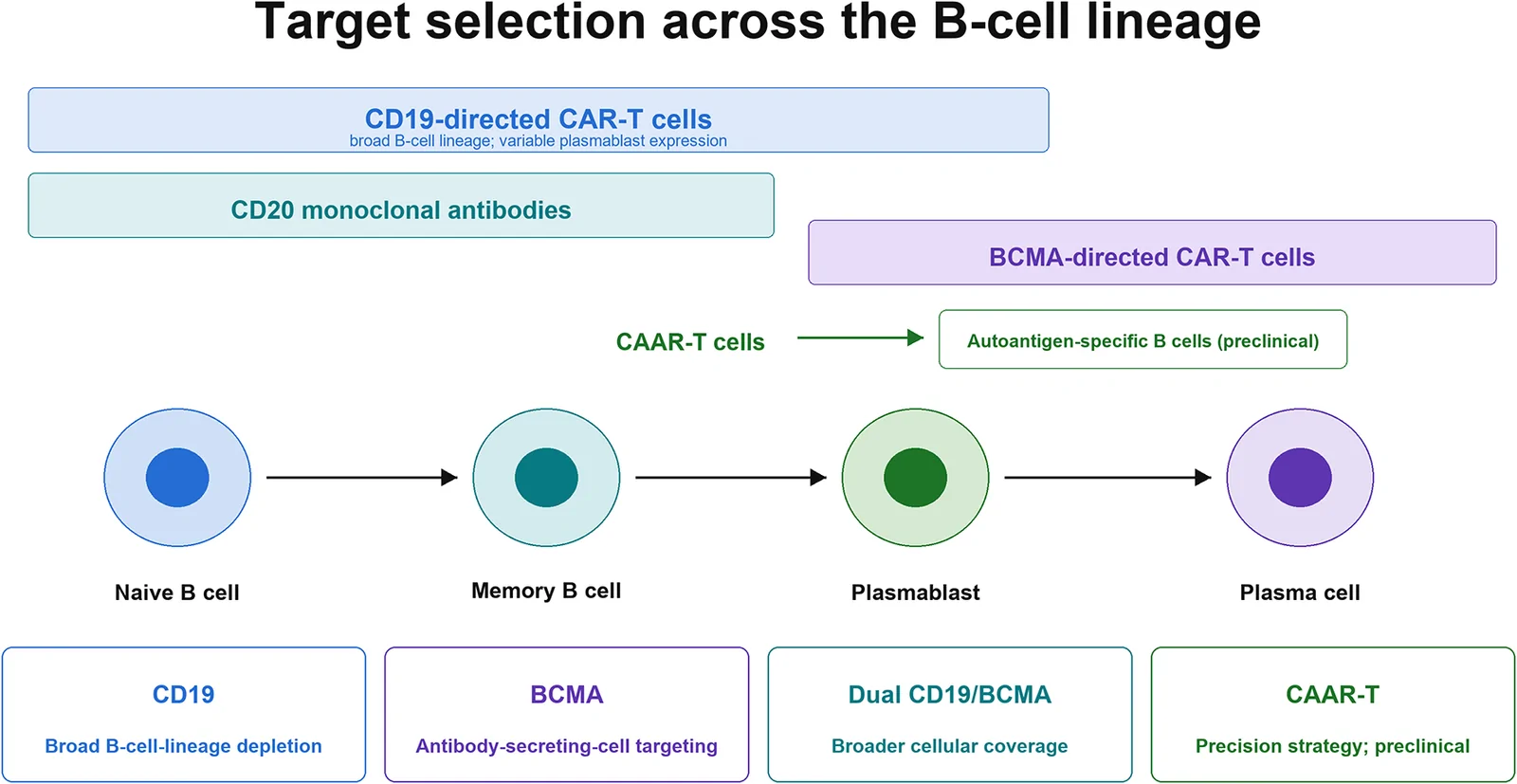
Target selection across the B-cell lineage. CD20 monoclonal antibodies are shown as a clinical comparator targeting mature B cells. CD19-directed CAR-T cells provide broader B-lineage coverage, although expression may be variable in plasmablasts; BCMA-directed CAR-T cells principally target plasmablasts and plasma cells. Dual CD19/BCMA-directed strategies aim to broaden lineage coverage, whereas CAAR-T cells are designed for autoantigen-specific depletion of autoreactive B cells and remain preclinical in neurologic disease.

**Table 1:** Evidence overview of CAR-T-cell therapy in major autoimmune neurologic diseases.

Disease	Candidate targets	Current evidence status	Prudent interpretation
Multiple sclerosis	CD19, BCMA	Anti-CD20 therapy establishes the B-cell axis; reports of CD19-directed CAR-T-cell therapy in two patients and a phase 1 BCMA study in five patients are available (22–28).	May be most relevant for highly active refractory disease or progressive disease with objective inflammation; B-cell depletion should not be equated with reversal of neurodegeneration.
NMOSD	BCMA; selected CD19	A phase 1 anti-BCMA CAR-T-cell study treated 12 AQP4-IgG-positive patients; 11 remained relapse-free at a median 5.5 months. All CRS events were grade 1–2 and infections occurred in 58% (29–34).	The uncontrolled design and short follow-up preclude comparative conclusions. Established targeted therapies set a high threshold for patient selection.
MOGAD	CD19	A refractory MOGAD case relapsed with optic neuritis on day 29 and subsequently remained relapse-free for approximately 1 year without maintenance immunotherapy (35–37).	This Class IV signal cannot establish efficacy; early relapse, phenotype heterogeneity and antibody dynamics require cautious interpretation.
Autoimmune encephalitis	CD19; BCMA as a candidate; CAAR-T preclinical	A treatment-refractory anti-DAGLA encephalitis case improved after CD19-directed CAR-T-cell therapy, with lower serum/CSF antibody levels and disappearance of CSF oligoclonal bands (38–44).	This single case cannot be generalized to other encephalitis subtypes; infection, ICANS and irreversible injury must be distinguished from active autoimmunity.
Myasthenia gravis	BCMA, CD19, dual CD19/BCMA; CAAR-T preclinical	Evidence includes CD19 case reports, phase 1b/2a and randomized phase 2b transient mRNA CAR-T-cell studies, and an early dual-target study (10, 11, 14–18, 45, 46).	This is the most mature neurologic evidence area, but antibody subtype and persistent versus transient platforms should be analyzed separately.
SPS/CIDP	CD19, BCMA	Individual CD19-directed CAR-T-cell reports are available in SPS, and a two-patient anti-BCMA study is available in CIDP (47–49).	Evidence remains uncontrolled. Selection should rely on objective activity, a plausible humoral mechanism and potentially reversible dysfunction.

AQP4-IgG, aquaporin-4 immunoglobulin G; BCMA, B-cell maturation antigen; CAAR-T, chimeric autoantibody receptor T cell; CAR-T, chimeric antigen receptor T cell; CIDP, chronic inflammatory demyelinating polyradiculoneuropathy; CRS, cytokine release syndrome; CSF, cerebrospinal fluid; DAGLA, diacylglycerol lipase alpha; ICANS, immune effector cell-associated neurotoxicity syndrome; MOGAD, myelin oligodendrocyte glycoprotein antibody-associated disease; mRNA, messenger RNA; NMOSD, neuromyelitis optica spectrum disorder; SPS, stiff-person syndrome.

This mechanistic framework should also guide immune monitoring. Baseline assessment should include B-cell and plasma-cell subsets, serum immunoglobulins, disease-relevant autoantibodies, infection screening, and measures of neurologic activity. Early and long-term follow-up should evaluate CAR-T-cell expansion and persistence, depletion and reconstitution of targeted immune populations, immunoglobulin recovery, toxicity, infection, and disease-specific outcomes (6, 19, 20). B-cell recovery alone should not trigger retreatment; additional therapy should be considered only when recurrent clinical or radiologic activity is accompanied by evidence of re-emerging pathogenic immunity. Combination strategies remain investigational. Current evidence does not support immune-checkpoint blockade as a routine adjunct; because it may aggravate autoimmunity, it should only be considered within a prospective controlled protocol (21). This caution is based on indirect safety evidence rather than studies of neuroimmunologic CAR-T-cell combinations.

## 3 Multiple sclerosis

Multiple sclerosis (MS) is one of the most frequently discussed but also most complex settings for CAR-T-cell translation. The efficacy of anti-CD20 therapy in relapsing MS and primary progressive MS established the importance of B cells in inflammatory activity and some aspects of progression (22, 23). Ofatumumab further reduced relapsing activity compared with teriflunomide, reinforcing B-cell depletion as a central therapeutic axis in MS (24). The longitudinal association between Epstein-Barr virus infection and MS risk supports a link between B cells and MS susceptibility (25). Clonally expanded B cells in MS can also bind EBV EBNA1 and GlialCAM, supporting a potential cross-reactive mechanism (26).

Nevertheless, the efficacy of anti-CD20 therapy does not imply that CAR-T cells will necessarily be superior to existing therapies. MS, particularly progressive MS, involves chronic active lesions, meningeal inflammation, myeloid activation, and neurodegenerative change, not all of which are fully dependent on peripheral B cells. A 2024 report of CD19-targeted CAR-T-cell therapy in two patients with MS provided feasibility and preliminary safety signals, but the sample size was too small to assess efficacy (27). A 2025 early study of BCMA-directed CAR-T-cell therapy in progressive MS introduced a plasma-cell- and central-immune-remodeling hypothesis and reported patient-level clinical and immunologic observations (28). Randomized studies and longer follow-up are required to distinguish treatment effects from natural fluctuation and irreversible progression. The hypothesized advantage over anti-CD20 monoclonal antibodies is deeper depletion of tissue-resident B-lineage cells; however, the absence of head-to-head studies and the contribution of non-B-cell mechanisms preclude claims of clinical superiority (7, 22–28).

CAR-T-cell research should not enroll unselected patients with relapsing MS. More rational candidates may include patients with highly active disease despite high-efficacy disease-modifying therapy and patients with progressive disease who retain clear evidence of inflammatory activity. Endpoints should extend beyond annualized relapse rate. They should include new or enlarging MRI lesions, markers of chronic active lesions, cerebrospinal fluid oligoclonal bands or free light chains, serum neurofilament light chain, GFAP, cognition, fatigue, and long-term disability progression. Across the major candidate diseases, the present evidence landscape is summarized qualitatively in **Figure 3**. In trials, longitudinal assessment should combine B-cell reconstitution, immunoglobulins, MRI activity, NfL/GFAP, relapses, and disability. Retreatment should require renewed inflammatory activity rather than B-cell recovery alone (22–28).

**Figure 3:**
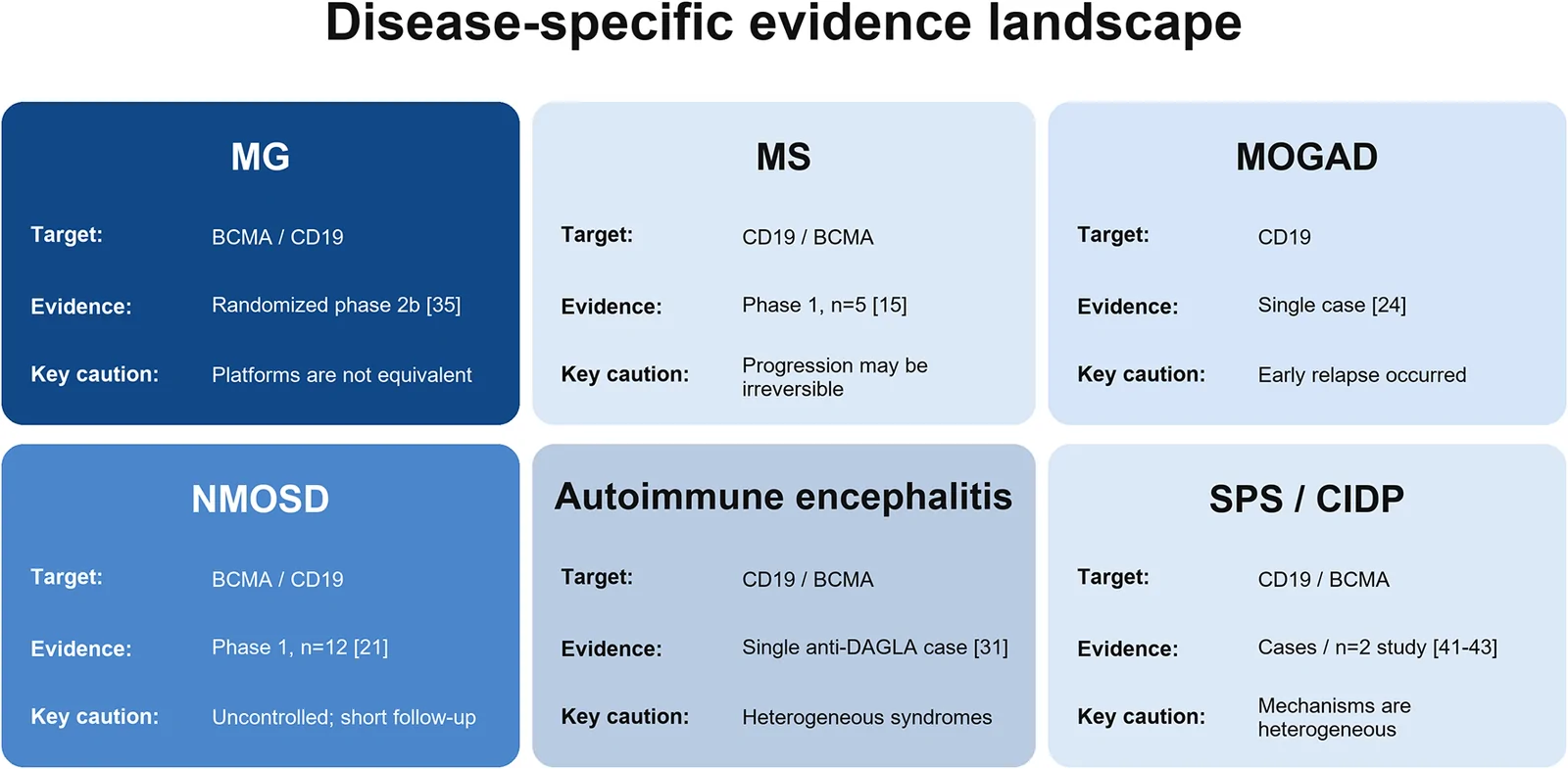
Disease-specific clinical evidence landscape. The figure summarizes the highest published level of human evidence, candidate target, and principal interpretive caution for MG, MS, MOGAD, NMOSD, autoimmune encephalitis, SPS, and CIDP. It is a qualitative synthesis and should not be interpreted as a comparative efficacy ranking.

## 4 Neuromyelitis optica spectrum disorder

Neuromyelitis optica spectrum disorder (NMOSD) is a prototypic antibody-mediated inflammatory disease of the central nervous system. The discovery of AQP4-IgG distinguished NMOSD from MS (29), and subsequent work established the roles of AQP4-IgG, astrocytic injury, complement activation, and granulocytic inflammation (30). Several mechanistically defined treatments now have pivotal or approval-level evidence, including the C5 complement inhibitor eculizumab (31), the anti-CD19 monoclonal antibody inebilizumab (32), and the IL-6 receptor blocker satralizumab (33).

NMOSD provides a biologically clear antibody-disease model for CAR-T-cell therapy, but the presence of highly effective established treatments raises the threshold for clinical use. A single-arm phase 1 study of BCMA-directed CAR-T-cell therapy in 12 patients with relapsed or refractory AQP4-IgG-seropositive NMOSD reported no relapse in 11 patients during a median follow-up of 5.5 months; however, all patients experienced grade 1–2 CRS and 58% developed infections (34). These uncontrolled interim findings support feasibility but do not establish comparative efficacy. In theory, CD19-directed CAR-T cells could produce deeper B-lineage depletion than inebilizumab, and BCMA-directed CAR-T cells could directly target AQP4-IgG-producing plasma cells. In practice, CAR-T-cell therapy should be investigated primarily in extremely refractory, frequently relapsing patients who have failed or cannot tolerate existing targeted therapies. Because NMOSD relapses can cause irreversible visual or spinal cord disability, trial design must give equal priority to relapse prevention, early rescue plans, antibody titers, and infection risk. No study has demonstrated superiority over inebilizumab or complement- or IL-6-pathway blockade (31–34). Proposed monitoring should integrate relapse, AQP4-IgG, visual and spinal function, B-cell and immunoglobulin recovery, and infection. Repeat therapy should require objective recurrence with evidence of renewed pathogenic immunity (29–34).

## 5 MOG antibody-associated disease

Myelin oligodendrocyte glycoprotein antibody-associated disease (MOGAD) differs from both AQP4-positive NMOSD and MS. Its clinical phenotypes include optic neuritis, myelitis, ADEM-like presentations, and brainstem or cortical encephalitis. A 2021 disease-spectrum review and the 2023 international diagnostic criteria clarified the importance of MOG-IgG testing, core clinical phenotypes, and exclusion criteria (35, 36). The relationship between antibody status and disease activity is often more direct in MOGAD than in MS, but major differences exist among pediatric, adult, monophasic, and relapsing courses.

One of the most direct neuroimmunologic CAR-T-cell reports to date concerns refractory MOGAD. In a 2024 report, a patient treated with CD19-directed CAR-T cells had an early optic-neuritis relapse on day 29, followed by approximately 1 year without further relapse or maintenance immunotherapy (37). The value of this case lies in showing that the treatment workflow is feasible in a central demyelinating antibody disease and that deep B-cell depletion may alter relapse trajectories. However, a single case cannot exclude regression to the mean, residual effects of prior immunotherapy, or the natural variability of the disease. Future studies should prioritize patients with repeated relapses, failure of prior B-cell depletion or maintenance therapy, and high-risk features between attacks. Because CAR-T platforms differ in the B-lineage compartments they target, **Table 2** compares the main candidate strategies, their expected advantages, and their principal limitations. No direct comparison with existing maintenance immunotherapy is available, and this observation should remain hypothesis generating (35–37). Follow-up should include relapse, MOG-IgG, visual and spinal outcomes, B-cell reconstitution, immunoglobulins, and infection (35–37).

**Table 2:** Candidate CAR-T-cell strategies for neuroimmunologic diseases.

Strategy	Target-cell range	Potential advantages	Main limitations
CD19-directed CAR-T cells (1, 2, 6, 7)	Pre-B cells through memory B cells, with variable expression on some plasmablasts; mature long-lived plasma cells are usually spared	Broad and potentially deeper B-cell-lineage depletion than CD20 monoclonal antibodies	Hypogammaglobulinemia, infection, prolonged B-cell aplasia and nonspecific immune loss
BCMA-directed CAR-T cells (10, 11, 28, 34, 49)	Plasmablasts and plasma cells	More directly targets sustained autoantibody production	May spare antigen-presenting B cells; plasma-cell depletion increases infection and immunoglobulin concerns
Dual CD19/BCMA CAR-T cells (14)	B-cell lineage plus antibody-secreting cells	Broader cellular coverage through a second CAR or dual-target construct	Toxicity, manufacturing and immune-reconstitution effects may be more complex
Transient mRNA CAR-T cells (10, 11)	Determined by the encoded CAR target; transient expression	Controllable and repeatable; may avoid long-term CAR persistence	Requires repeat dosing; depth and durability differ from persistent products
CAAR-T cells (16–18)	Autoantigen-specific B cells	Potentially preserves nonpathogenic B cells and is more precise	Epitope and antigen-conformation heterogeneity, soluble-antibody interference and limited clinical evidence

BCMA, B-cell maturation antigen; CAAR-T, chimeric autoantibody receptor T cell; CAR-T, chimeric antigen receptor T cell; mRNA, messenger RNA.

## 6 Autoimmune encephalitis

Autoimmune encephalitis is among the most complex potential applications of CAR-T-cell therapy. The discovery of anti-NMDAR encephalitis and studies of antibody-mediated pathogenicity demonstrated that antibodies against neuronal surface antigens can cause reversible synaptic dysfunction and severe clinical syndromes (38). Large cohort data indicate that early immunotherapy, tumor treatment when relevant, and second-line therapy are associated with long-term outcomes (39). The 2016 clinical diagnostic framework emphasized that treatment should not wait for all antibody results but should integrate subacute psychiatric or behavioral changes, seizures, memory deficits, cerebrospinal fluid, MRI, and EEG evidence (40).

Mechanistically, anti-NMDAR encephalitis and other neuronal surface antibody diseases may be candidates for B-cell or plasma-cell targeting because antibody-mediated pathology, relapse, and refractory cases are well recognized (41–43). Human evidence now includes a single anti-DAGLA antibody-associated encephalitis case treated with CD19-directed CAR-T cells, with clinical improvement and reductions in serum and CSF antibody levels (44). This observation is preliminary and cannot establish efficacy across heterogeneous autoimmune encephalitides. Key risks include poor tolerance of lymphodepletion during acute critical illness and difficulty distinguishing CAR-T-related fever or neurotoxicity from worsening encephalitis. Further challenges include simultaneous tumor management in paraneoplastic cases and incomplete coverage of intrathecal antibody production by peripheral B-cell depletion. In the near term, CAR-T cells are best viewed as investigational rescue therapy for highly refractory cases rather than routine early second-line treatment. No head-to-head study has compared CAR-T cells with rituximab or other second-line immunotherapies, and the present rationale remains mechanistic (39–44). Disease-specific monitoring should include cognition and psychiatric status, seizures, CSF, MRI and EEG activity, antibody dynamics, and tumor status when relevant (38–44).

## 7 Myasthenia gravis and neuromuscular junction disorders

Myasthenia gravis (MG) is currently the autoimmune neurologic disease in which CAR-T-cell evidence is growing most rapidly. International consensus guidance has established a tiered management framework involving cholinesterase inhibitors, glucocorticoids, conventional immunosuppressants, thymic intervention, immunoglobulin or plasma exchange, and newer targeted therapies (45). The unmet need in refractory MG includes chronic immunosuppressive burden, myasthenic crisis risk, symptom fluctuation, and persistent autoantibody production.

A case report of CD19-directed CAR-T-cell therapy for refractory MG suggested that deep B-cell depletion may improve clinical status in a highly refractory patient (46). The mRNA CAR-T-cell platform is particularly relevant for clinical translation. The MG-001 open-label phase 1b/2a study evaluated the safety and clinical activity of autologous mRNA CAR-T-cell therapy in MG (10). A subsequent randomized, double-blind, placebo-controlled phase 2b trial of BCMA-directed mRNA CAR-T-cell therapy moved this strategy to a higher level of evidence (11). In addition, a single-arm phase 1 trial of anti-BCMA/CD19 CAR-T-cell therapy explored simultaneous targeting of B cells and plasma cells (14). A report of MG with concomitant Lambert-Eaton myasthenic syndrome suggested that broad B-cell depletion may also affect complex neuromuscular junction autoimmune syndromes (15). CAR-T cells may offer a treatment-free interval after a finite treatment course, but current studies do not establish superiority over established targeted therapies (10, 11, 14, 45, 46).

Another important MG strategy is antigen-specific CAAR-T-cell therapy. The first autoimmune CAAR-T concept came from pemphigus models, in which autoantigen domains were used to selectively eliminate autoreactive B cells (16). In MG, MuSK CAAR-T-cell studies demonstrated precision targeting of MuSK-specific B cells (17), and AChR CAAR-T-cell work further explored antigen-specific depletion for AChR antibody-positive MG (18). Compared with CD19 or BCMA CAR-T cells, the theoretical advantage of CAAR-T cells is preservation of most normal B-cell immunity. Major challenges include autoantigen conformation, antibody heterogeneity, epitope coverage, and manufacturing complexity. Monitoring should integrate MG-ADL/QMG, respiratory function, AChR or MuSK antibody levels, targeted-cell depletion and reconstitution, immunoglobulins, and infection. Retreatment should require objective clinical worsening with supportive immunologic evidence (10, 11, 14, 17, 18, 45, 46).

## 8 Stiff-person syndrome, CIDP, and other peripheral neuroimmune diseases

Stiff-person syndrome (SPS) is often associated with antibodies against GAD65, glycine receptor, amphiphysin, or related targets. It can be highly disabling, and some patients respond inadequately to conventional immunotherapy. A 2024 report of successful anti-CD19 CAR-T-cell therapy in severe treatment-refractory SPS suggested that broad B-cell depletion may benefit selected refractory cases (47). A 2025 case of advanced SPS with concomitant MG further suggested that CD19 CAR-T-cell therapy may produce cross-phenotype signals in patients with multiple antibody-associated syndromes (48). These reports remain uncontrolled and do not establish superiority over conventional immunotherapy (47, 48). Follow-up should distinguish symptomatic fluctuation from sustained functional improvement and track relevant antibodies, B-cell recovery, immunoglobulins, and infection (47, 48).

Chronic inflammatory demyelinating polyradiculoneuropathy (CIDP) has traditionally been considered an immune-mediated demyelinating peripheral nerve disease, usually treated with IVIG, glucocorticoids, or plasma exchange. Recent work on autoantibodies and nodal or paranodal antigens has made some CIDP subtypes more clearly antibody mediated. A 2025 report of anti-BCMA CAR-T-cell therapy in relapsed or refractory CIDP provided preliminary human observations (49). The key question is how to identify patients whose disease is truly maintained by plasma cells and autoantibodies, and how to distinguish treatment response from axonal injury, long-term disability, and delayed clinical recovery. No comparison with IVIG, glucocorticoids, plasma exchange, or other targeted approaches is available (49). Retreatment decisions should be based on objective neurologic deterioration with evidence of renewed antibody-secreting-cell activity, rather than immune-cell recovery alone.

## 9 Safety, monitoring, and clinical-trial design

CAR-T-cell therapy for neuroimmune diseases must address two layers of neurologic risk. Patients already have central or peripheral nervous system injury. In addition, CAR-T cells can cause cytokine release syndrome (CRS) and immune effector cell-associated neurotoxicity syndrome (ICANS). The ASTCT consensus provides a unified grading framework for CRS and neurologic toxicity (19). Studies in oncologic CAR-T-cell therapy indicate that endothelial activation, blood-brain barrier disruption, inflammatory cytokines, and coagulation or vascular factors may contribute to neurotoxicity (50). Clinical and biologic correlation studies also suggest that fever, cytokines, T-cell expansion, and disease burden may be associated with neurotoxicity risk (51). Toxicity-management reviews emphasize that CAR-T-cell programs require predefined strategies for recognition, grading, supportive care, anti-cytokine therapy, and glucocorticoid use (20).

Neuroimmunologic trials should add disease-specific neurologic assessments to the oncology CAR-T safety framework. Baseline evaluation should include neurologic scales, MRI, cerebrospinal fluid measures, serum autoantibodies, immunoglobulins, infection screening, vaccination status, and prior immunotherapy exposure. MS trials may incorporate EDSS, MRI activity, NfL/GFAP, and relapse endpoints. NMOSD and MOGAD trials should emphasize time to first relapse, visual or spinal cord function, and AQP4 or MOG antibody dynamics. MG studies require MG-ADL, QMG, crisis events, and respiratory function. Autoimmune encephalitis trials should include modified Rankin Scale, cognitive and psychiatric assessments, seizure control, and cerebrospinal fluid markers. Follow-up should extend beyond 3–6 months because B-cell reconstitution, hypogammaglobulinemia, infection, secondary autoimmunity, and long-term neurologic outcomes require observation over years. A phase-based safety monitoring framework is illustrated in **Figure 4**. Early post-infusion assessment should document CAR-T-cell expansion, depletion of targeted immune populations, fever, inflammatory markers, infection, and new neurologic deficits (19, 20, 50, 51).

**Figure 4:**
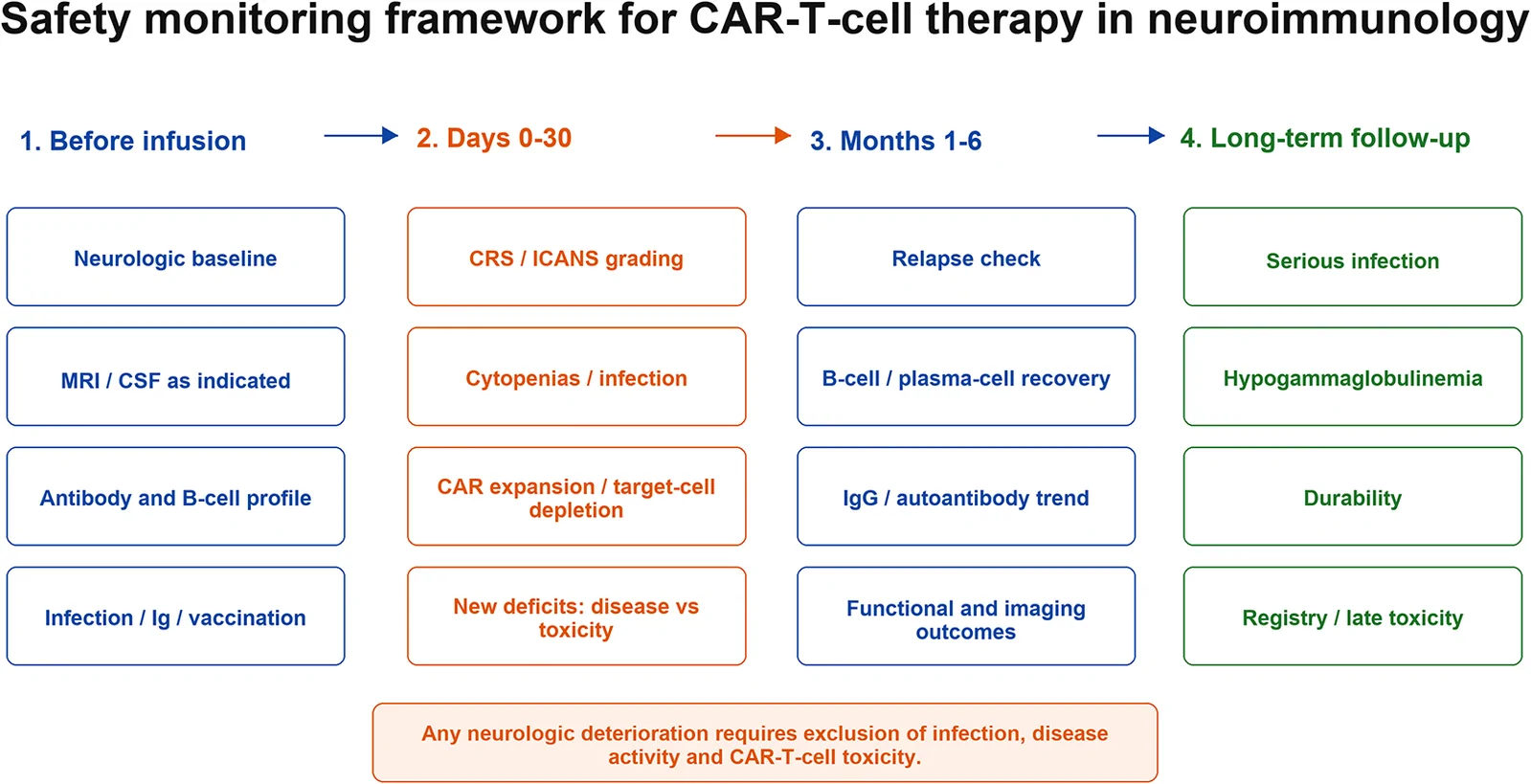
Phase-based safety and immune-monitoring framework for CAR-T-cell therapy in neuroimmunology. Early assessment includes CRS/ICANS, cytopenias and infection, CAR-T-cell expansion and target-cell depletion, and evaluation of new neurologic deficits as disease activity versus treatment toxicity. Intermediate and long-term follow-up address relapse, B-cell/plasma-cell recovery, immunoglobulin and autoantibody trends, functional and imaging outcomes, delayed infection, hypogammaglobulinemia, secondary autoimmunity or malignancy, disability, quality of life, and registry-based outcomes.

Patient selection is central to the risk-benefit balance. In diseases with effective and safe established therapies, CAR-T cells should not be moved forward casually. In patients with severe, relapsing, organ-threatening disease that has failed or cannot tolerate conventional targeted therapies, CAR-T-cell therapy may be a rational investigational option. Trials must define rescue therapy, infection prophylaxis, immunoglobulin replacement, pregnancy management, vaccination, and long-term malignancy surveillance. For central nervous system diseases, an independent neurologic safety committee should help distinguish primary disease activity, infection, metabolic encephalopathy, drug effects, and CAR-T-related neurotoxicity. Given the neurologic vulnerability of the intended population, **Table 3** outlines baseline assessments, early post-infusion monitoring, long-term endpoints, rescue or adjunctive therapy, and proposed criteria for additional courses in neuroimmunologic CAR-T studies.

**Table 3:** Safety monitoring, rescue therapy, and retreatment criteria for CAR-T-cell studies in autoimmune neurologic diseases.

A. Immune and safety monitoring					
Domain	Baseline assessment		Early immune and safety monitoring		Long-term immune studies/outcomes
General CAR-T-cell safety	CBC and organ function; infection screening; vaccination; immunoglobulins; lymphocyte subsets and B/plasma-cell phenotype; autoantibody; prior immunotherapy; reversible versus fixed injury (6, 19, 20, 50, 51).		CRS/ICANS grade; cytopenias; infection; CAR expansion; target-cell depletion; immunoglobulins and autoantibody trends; attribution of new neurologic deficits.		CAR persistence; B/plasma-cell reconstitution; immunoglobulin and vaccine-specific immunity; autoantibody; late cytopenia, infection, secondary autoimmunity and malignancy.
MS	EDSS; relapse and MRI activity; serum NfL/GFAP; prior high-efficacy therapy; CSF immune measures when indicated; evidence of active inflammation.		Neurologic examination; fever/infection and pseudo-relapse exclusion; MRI/CSF if new deficits occur; CAR kinetics and B-cell depletion.		Relapse, MRI lesions, EDSS, NfL/GFAP, cognition and fatigue; CAR persistence and B/plasma-cell recovery.
NMOSD	AQP4-IgG; relapse history; visual/spinal function; MRI; prior targeted therapy; B/plasma-cell profile and infection risk.		Frequent opticospinal assessment; attack versus infection/ICANS; cytopenias, CAR kinetics, target-cell depletion and AQP4-IgG trend.		Time to relapse, disability, AQP4-IgG, immunoglobulins, B/plasma-cell recovery, infection and treatment-free control.
MOGAD	MOG-IgG; monophasic versus relapsing course; prior optic neuritis/myelitis/ADEM; visual/spinal function; MRI and maintenance-therapy failure.		Early relapse surveillance; disease versus infection/ICANS; CAR kinetics, B-cell depletion, immunoglobulin and MOG-IgG trend.		Time to relapse, visual/spinal outcome, MOG-IgG, B-cell recovery, steroid exposure and treatment-free control.
Myasthenia gravis	MG-ADL and QMG/MGC; respiratory/bulbar function; AChR/MuSK subtype; crisis/rescue dependence; thymic status and current therapy.		Respiratory/bulbar monitoring; infection versus crisis; CRS/ICANS; CAR kinetics, B/plasma-cell depletion, immunoglobulin and autoantibody trend.		Minimal symptom expression, crisis-free survival, rescue use, steroid taper, autoantibody, immunoglobulin and immune reconstitution.
Autoimmune encephalitis	mRS/CASE; cognition/psychiatry; seizures and EEG; MRI/CSF; serum/CSF antibodies; infection/tumor screening; active inflammation versus fixed injury.		EEG when indicated; infection/metabolic workup; ICANS differentiation; autonomic/respiratory monitoring; CAR kinetics and CSF/antibody changes.		Relapse, function, cognition, seizure freedom, MRI/CSF, antibodies/OCBs, CAR persistence and immune recovery.
SPS/CIDP	Objective stiffness/spasm or INCAT/I-RODS/MRC measures; gait; electrophysiology for CIDP; serum/CSF antibodies; medication or IVIG dependence; irreversible axonal injury.		Neurologic, gait and respiratory monitoring; infection, cytopenias and CRS/ICANS; CAR kinetics, target-cell depletion and immunoglobulins.		Sustained function, medication/IVIG-free interval, antibody and B/plasma-cell recovery, infection and late toxicity.

B. Rescue therapy and proposed retreatment criteria					
Domain		Rescue/compatible adjuncts		Proposed criteria for an additional course	
General CAR-T-cell safety		Protocol-defined CRS/ICANS treatment; antimicrobials; immunoglobulin replacement; disease rescue with corticosteroids, IVIG or plasma exchange when indicated. Immune-checkpoint blockade is not routine outside a prospective protocol.		Objective recurrent disease plus concordant re-emergence of pathogenic immunity after infection, toxicity and fixed injury are excluded. B-cell recovery alone is insufficient.	
MS		Prespecified corticosteroid and plasma-exchange pathway for an objective inflammatory relapse.		A confirmed inflammatory relapse or new MRI activity with mechanistic immune reactivation; progression without inflammation is not a retreatment trigger.	
NMOSD		Immediate high-dose corticosteroids for a confirmed attack, with plasma exchange when indicated.		Objective optic neuritis or myelitis together with renewed pathogenic humoral activity; antibody or B-cell return alone is insufficient.	
MOGAD		Corticosteroids, IVIG or plasma exchange according to the attack phenotype.		A confirmed recurrent inflammatory event with concordant immune reactivation; isolated seropositivity or B-cell recovery is insufficient.	
Myasthenia gravis		Predefined FVC/bulbar thresholds for IVIG/plasma exchange and intensive care; symptomatic therapy may continue as specified.		Objective recurrent weakness plus re-emerging pathogenic immunity. Repeat transient mRNA dosing follows protocol; persistent-product retreatment requires separate review.	
Autoimmune encephalitis		ICU, antiseizure and disease-specific immunotherapy pathways for status epilepticus, autonomic instability or clinical deterioration.		Objective recurrent inflammatory encephalitis with concordant CSF/antibody or B-cell evidence; progressive fixed injury alone is insufficient.	
SPS/CIDP		Symptomatic therapy, IVIG, corticosteroids or plasma exchange according to the disease phenotype.		Objective recurrent activity plus a plausible renewed humoral mechanism; GAD65 titer, antibody persistence or B-cell return alone is insufficient.	

AChR, acetylcholine receptor; ADEM, acute disseminated encephalomyelitis; AQP4-IgG, aquaporin-4 immunoglobulin G; CAR, chimeric antigen receptor; CASE, Clinical Assessment Scale in Autoimmune Encephalitis; CBC, complete blood count; CIDP, chronic inflammatory demyelinating polyradiculoneuropathy; CRS, cytokine release syndrome; CSF, cerebrospinal fluid; EDSS, Expanded Disability Status Scale; EEG, electroencephalography; FVC, forced vital capacity; GAD65, glutamic acid decarboxylase 65; GFAP, glial fibrillary acidic protein; ICANS, immune effector cell-associated neurotoxicity syndrome; ICU, intensive care unit; INCAT, Inflammatory Neuropathy Cause and Treatment; I-RODS, Inflammatory Rasch-built Overall Disability Scale; IVIG, intravenous immunoglobulin; MG, myasthenia gravis; MG-ADL, Myasthenia Gravis Activities of Daily Living; MGC, Myasthenia Gravis Composite; MOG-IgG, myelin oligodendrocyte glycoprotein immunoglobulin G; MOGAD, MOG antibody-associated disease; MRC, Medical Research Council; MRI, magnetic resonance imaging; mRS, modified Rankin Scale; MS, multiple sclerosis; MuSK, muscle-specific tyrosine kinase; NfL, neurofilament light chain; NMOSD, neuromyelitis optica spectrum disorder; OCBs, oligoclonal bands; QMG, Quantitative Myasthenia Gravis score; SPS, stiff-person syndrome.

Retreatment should not be triggered by B-cell recovery alone. It should require recurrent clinical or radiologic activity together with re-emergence of pathogenic immunity. Relapse despite persistent target-cell aplasia should prompt reconsideration of the target or disease mechanism rather than automatic repeat infusion. Disease-specific bridging therapy, including corticosteroids, IVIG, or plasma exchange, may be used when necessary, but its timing relative to lymphodepletion and CAR-T-cell expansion should be predefined. Current evidence does not support immune-checkpoint blockade as a routine adjunct; because it may aggravate autoimmunity, it should only be considered within a prospective controlled protocol (21).

## 10 Discussion

The value of CAR-T-cell therapy in autoimmune neurologic diseases is not simply to replace existing monoclonal antibodies, but to offer a possible deep immune-reconstituting strategy for a small subset of highly refractory patients. The strongest neurologic evidence is currently in MG, where open-label and randomized studies are available. MS has a small phase 1 study, NMOSD an uncontrolled phase 1 study, and MOGAD, autoimmune encephalitis, SPS, and CIDP remain supported mainly by individual cases or other early observations. Thus, efficacy conclusions cannot be generalized across diseases; only platform experience and safety-monitoring principles can be shared.

Future research has four priorities. First, enrollment criteria should be mechanism based rather than diagnosis based. Autoantibody positivity, recent inflammatory activity, prior response to B-cell or plasma-cell targeting, cerebrospinal fluid immune activity, and reversibility of tissue injury should all inform decision-making. Second, target and platform selection should be compared using the mechanistic framework in **Figure 2** and the disease-specific evidence in **Table 1**.

Third, neurologic endpoints must be disease specific. Antibody reduction or B-cell aplasia alone cannot prove neurologic benefit; functional scales, imaging, relapse measures, quality of life, and long-term disability are essential. Fourth, long-term risk requires special attention. Patients with autoimmune diseases often have long life expectancy, and the therapeutic goal is not short-term tumor control but durable safety and improved quality of life. These considerations can be operationalized as the proposed trial-design logic shown in **Figure 5**.

**Figure 5:**
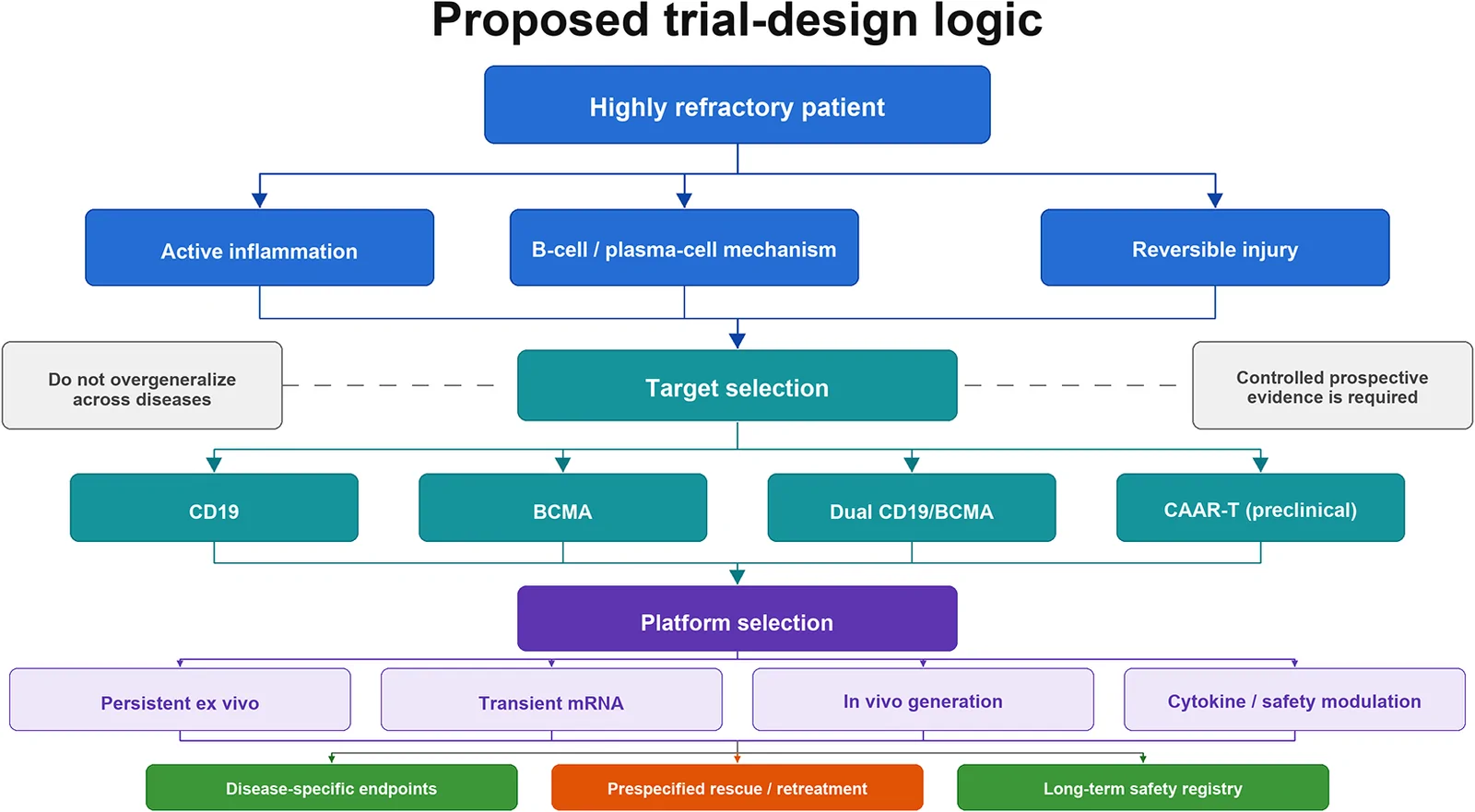
Proposed trial-design logic for CAR-T-cell therapy in autoimmune neurologic diseases. The framework links selection of highly refractory patients with active inflammation, a B-cell or plasma-cell-driven mechanism, and potentially reversible injury to disease-matched target selection and platform selection. It also incorporates disease-specific endpoints, predefined rescue and retreatment criteria, and long-term registry follow-up, while emphasizing the need for controlled evidence.

Before routine clinical use, major unresolved challenges include reproducible patient-selection criteria, manufacturing access and cost, long-term infection and hypogammaglobulinemia, secondary malignancy surveillance, and direct comparison with established targeted therapies. Controlled prospective studies with disease-specific endpoints are essential (6, 19, 20, 50, 51).

Overall, CAR-T-cell therapy opens a path from chronic immunosuppression toward immune reconstitution and potentially durable disease control in autoimmune neurologic diseases. That path remains narrow. It requires precise disease stratification, rigorous ethics oversight, long-term registry studies, and multidisciplinary care. At present, the most balanced conclusion is that CAR-T cells may be considered an investigational option for highly refractory autoimmune neurologic diseases with clear B-cell or plasma-cell-driven mechanisms. Their transition into routine care will depend on larger controlled studies and long-term real-world safety data.
